# Comparing analysis methods for quantification of myocardial oedema in patients following reperfused ST-elevation MI

**DOI:** 10.1186/1532-429X-13-S1-M11

**Published:** 2011-02-02

**Authors:** Tom Burchell, Andrew S Flett, Steffen E Petersen, L Ceri Davies, Saidi A Mohiddin, Anthony Mathur, Mark A Westwood

**Affiliations:** 1 London Chest Hospital, London, UK; 2 The Heart Hospital, London, UK

## Background

Myocardial oedema has been described as a marker of prognosis in ischaemic heart disease and CMR is able to characterise this using T2-weighted sequences. The inherent low signal to noise ratio of these sequences and low contrast between normal and abnormal myocardium makes quantification technically challenging. We therefore compared 5 methods of analysis in patients following reperfused ST-elevation MI.

## Methods

9 patients with successfully revascularised ST-elevation MI were recruited and were scanned on days 1, 3, 10, 20 and 90 following their PPCI with a 1.5T Philips Achieva (Philips Medical Systems). Images were obtained as continuous short-axis stacks covering the left ventricle (slice thickness 8mm, gap 2mm). Myocardial oedema was assessed using T2-weighted triple inversion turbo spin echo STIR imaging (TE 80, TR 1667). Image analysis was performed using dedicated software, CMR42 (Circle CVI, Calgary, Canada). Segmentation of the LV was performed by manually drawing endocardial and epicardial contours followed by semi-automated selection of normal remote myocardium per slice. Myocardial oedema was then calculated as >2SD and >3SD from normal remote myocardium for each slice (2SD and 3SD). For each scan the slice with the best contrast between normal and oedematous myocardium was chosen manually and either >2SD and >3SD was used to obtain a threshold level, which was applied to all other slices (2SD manual and 3SD manual). Finally a semi-automated method using the Otsu histogram comparison algorithm was used to quantify oedema (Otsu). All values are expressed as a percentage of the LV-mass.

## Results

**Figure 1:**
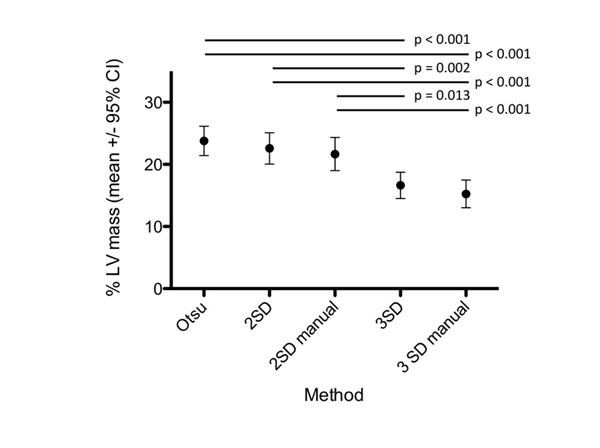


There was no difference between either 2SD and 2SD manual (22.6 ± 8.4 vs. 21.7 ± 8.9 p=ns) or 3SD and 3SD manual (16.6 ± 7.0 vs. 15.3 ± 7.4 p=ns). There was no difference between Otsu and either 2SD (23.8 ± 7.8 vs. 22.6 ± 8.4 p=ns) or 2SD manual (23.8 ± 7.8 vs. 21.7 ± 8.9 p=ns). There was a significant difference between Otsu and both 2SD methods compared with both 3SD methods (p-values <0.013), see Figure 1.

## Conclusions

There is no difference between the Otsu and both 2SD methods of myocardial oedema quantification in patients following reperfused MI. All 3 methods identify a larger volume of oedema than either of the 3SD methods. In future, the automated Otsu method could be used to replace the traditional >2SD method, potentially simplifying and shortening analysis times.

